# Altered sensory-weighting mechanisms is observed in adolescents with idiopathic scoliosis

**DOI:** 10.1186/1471-2202-7-68

**Published:** 2006-10-19

**Authors:** Martin Simoneau, Pierre Mercier, Jean Blouin, Paul Allard, Normand Teasdale

**Affiliations:** 1 Faculté de médecine, Division de kinésiologie, Université Laval, Québec, Canada; 2 Clinique d'orthopédie infantile de Québec and Département de Chirurgie, Université Laval, Québec, Canada; 3 UMR Mouvement & Perception CNRS et Université de la Méditerranée, Marseille, France; 4 Département de kinésiologie, Université de Montréal, Montréal, Canada

## Abstract

**Background:**

Scoliosis is the most common type of spinal deformity. In North American children, adolescent idiopathic scoliosis (AIS) makes up about 90% of all cases of scoliosis. While its prevalence is about 2% to 3% in children aged between 10 to 16 years, girls are more at risk than boys for severe progression with a ratio of 3.6 to 1. The aim of the present study was to test the hypothesis that idiopathic scoliosis interferes with the mechanisms responsible for sensory-reweighting during balance control.

**Methods:**

Eight scoliosis patients (seven female and one male; mean age: 16.4 years) and nine healthy adolescents (average age 16.5 years) participated in the experiment. Visual and ankle proprioceptive information was perturbed (eyes closed and/or tendon vibration) suddenly and then returned to normal (eyes open and/or no tendon vibration). An AMTI force platform was used to compute centre of pressure root mean squared velocity and sway density curve.

**Results:**

For the control condition (eyes open and no tendon vibration), adolescent idiopathic scoliosis patients had a greater centre of pressure root mean squared velocity (variability) than control participants. Reintegration of ankle proprioception, when vision was either available or removed, led to an increased centre of pressure velocity variability for the adolescent idiopathic scoliosis patients whereas the control participants reduced their centre of pressure velocity variability. Moreover, in the absence of vision, adolescent idiopathic scoliosis exhibited an increased centre of pressure velocity variability when ankle proprioception was returned to normal (i.e. tendon vibration stopped). The analysis of the sway density plot suggests that adolescent idiopathic scoliosis patients, during sensory reintegration, do not scale appropriately their balance control commands.

**Conclusion:**

Altogether, the present results demonstrate that idiopathic scoliosis adolescents have difficulty in reweighting sensory inputs following a brief period of sensory deprivation.

## Background

Scoliosis is the most common type of spinal deformity. In North American children, adolescent idiopathic scoliosis (AIS) makes up about 90% of all cases of scoliosis [1]. While its prevalence is about 2% to 3% in children aged between 10 to 16 years, girls are more at risk than boys for severe progression with a ratio of 3.6 to 1.

Biomechanical factors such as three-dimensional deviation of the spine are likely to lead to balance control problems. Morphologic changes associated with scoliosis alter the orientation of the head, shoulders, scapula and pelvis in all three planes [2]. These postural changes in body attitude associated with scoliosis could be responsible for the balance problems that have been reported in AIS [3].

A small body deviation from a perfect vertical position needs a corrective torque exerted by the lower limbs to counteract the destabilization. A widely held view is that the corrective torque is generated through the action of active feedback-control mechanisms based on information about body motion. This information would be conveyed by the visual [4-7], proprioceptive [8-13] and vestibular [14-17] systems.

Although there is no established cause, idiopathic scoliosis has been associated with several sensory and motor impairments. These include neurogenic disorder of paraspinal muscles as measured by myotatic stretch reflex responses [18], deficit at the cerebral level [19], imbalance between the resting firing frequency between the two peripheral vestibular end organs [20,21], muscular imbalance between both sides of the spine [22-24], proprioceptive disorders [25-27] and asymmetries in the ventral pons or brainstem dysfunction [28,29]. These impairments would lead to balance control problems [30-33].

Deficits in the structure and functioning of peripheral systems are vast in AIS. A lesion of the posterior column pathways has been suggested as a possible cause of scoliosis. In animal studies, scoliosis has been induced by damaging the posterior column pathway at the dorsal root as well as in the thoracic cord [34,35]. Theses observations led some researchers to investigate whether patients with idiopathic scoliosis would respond differently to healthy control participants to a mechanical stimulus [30,36-38]. No consensus, however, has been reached. For example, Wyatt et al. (1986) and Barrack et al. [37] found that AIS patients had a lower proprioceptive threshold (more sensitive) whereas McInnes et al. [39] reported that the AIS group had a significantly higher vibratory threshold (less sensitive) than healthy control participants. Responses to mechanical (e.g., vibration) stimulation provide a means of evaluating the threshold of the proprioceptive system; it does not determine the capability of the brain to transform sensory perception into appropriate motor responses. One way to assess the ability of the brain to transform available sensory inputs into appropriate motor commands is to manipulate sensory information and quantify its effect on balance control. Indeed, sensory deprivation in AIS patients has been considered to exacerbate body sway oscillations[31,40].

Herman et al. [19] reported that idiopathic scoliosis patients exhibit perceptual impairments, deficits in sensorimotor adaptation, learning and balance control. According to the authors, these deficits would be the signature of disorders at higher integrative levels of the central nervous system. Yet, the ability of adolescents with idiopathic scoliosis to adequately re-weight sensory inputs following sensory integration has not been investigated. One method to determine the ability of the brain to integrate sensory inputs consists of monitoring balance stability during transient sensory perturbations (e.g., no vision to vision). A decreased stability would result from a difficulty to dynamically re-weight the sensory inputs when a new input is made available following a period of deprivation. Indeed, when sensory information is added, the brain has to recalibrate the postural set based on the new sensory content. Therefore, decreased stability following sudden increase in sensory information would enable us to rule out a simple exclusive peripheral sensory problem interpretation.

Two non exclusive hypotheses could explain the balance control problems observed in AIS patients: a *biomechanical hypothesis *which gives importance to such factors as the shape of the trunk and the changes in the relationships between body segments and the trunk, and a *sensory integration hypothesis *which predicts impairments in the dynamic regulation of sensorimotor integration by the inappropriate weighting of sensory inputs. The present study tested the second hypothesis. We hypothesized that transient sensory manipulation would lead to inadequate balance control in AIS patients but not in healthy control participants suggesting that central mechanisms involved in multisensory integration are altered in AIS.

## Methods

### Subjects

Eight idiopathic scoliosis patients (seven female and one male; mean age 16.3 ± 2.1 years) participated in the experiment. Scoliosis patients had been screened and diagnosed by a pediatric orthopedic surgeon (one of the authors, P. Mercier). None of the scoliosis had abnormal neurological sign. We did not use magnetic resonance imaging (MRI). In a study, with a larger number of patients (n = 1280), only 2% showed abnormal findings [41]. Besides, none of our patients had an indicator necessitating MRI (see Table I and II of [41] for a list of indicator). All patients had right thoracic convex curve (mean Cobb angle of 45.6 ± 7.5°) and three patients had left lumbar convex compensatory curve (mean Cobb angle of 38.5 ± 4.5°). All patients had Riser sign greater than 3 and their curve had not progressed during the last year. We made no attempt to determine whether a specific curve pattern led to greater balance control problem. Briefly, the Cobb angle is defined by the relationship between two lines drawn parallel to the top and bottom of the vertebral bodies at the beginning and end of the curve. The Cobb angle is the angle between these two lines (or lines drawn perpendicular to them). A scoliotic curve exists when the angle measures at least 10 degrees. Most curves are considered significant if greater than 25–30°. Curves greater than 45–50° are considered severe and potentially harmful (e.g., lung problem). The patients were not under active treatment and none of them had had surgery. Brace treatment was recommended for three subjects to reduce the incidence of curve progression, but none of them had ever worn a brace. All spinal curves were right thoracic convex. Out of the eight thoracic curvatures, four had left lumbar compensatory curves (mean 39° standard deviation 4.2°). The control group consisted of nine adolescent females (average age of 16.5 ± 1.7 years). None of them reported any neurological or orthopedic problems. All participants and tutors gave their informed consent according to university protocols and the experiment was approved by the local ethics committee.

### Apparatus

An AMTI force platform (model OR6-6, Watertwon, USA) was used to determine the displacement of the center of pressure (CP). The force platform signals were sampled at 200 Hz using a 12-bit A/D converter. Ankle proprioception was perturbed by means of vibratory stimulation. As reported by Burke et al. [42,43], both human primary and secondary spindles endings respond to vibration stimuli. When applied to mutually antagonistic ankle muscles, the vibratory stimulus gives instant rise to a background noise and deprives subjects from relevant ankle proprioceptive information produced by body sway oscillations.

The vibration amplitude and frequency were 3 mm and 80 Hz. The vibrators were fixed to each subject's ankles, over the tendons of the soleus, gastrocnemius and tibialis anterior by means of rubber bands. The activation and deactivation of the vibrators were computer controlled. For convenience, we will herein refer to the tendon vibration condition as the perturbed proprioception interval. For the vision to no-vision or no-vision to vision intervals, participants closed their eyes when the computer released an auditory signal and re-opened their eyes when they heard it a second time.

### Tasks and procedures

**Figure 1:**
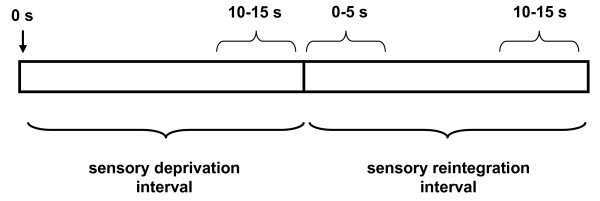
**Temporal sequencing across sensory deprivation and sensory reintegration interval**. A trial is made of a series of two 15 s intervals. The first interval, sensory deprivation, is performed under sensory deprivation (i.e. perturbed ankle proprioception and/or no vision). For the second interval (sensory reintegration interval), ankle proprioception and/or vision returned to normal. For the sensory deprivation interval, only the last 5 s epoch is used for data analysis. From the 15 s of the sensory reintegration interval, two epochs are selected. The first epoch represents the first 5 s whereas the second epoch concerns the last 5 s.

Subjects stood barefoot on the force platform with feet 10-cm apart and the arms along the body. They maintained an upright posture while fixating on a small target in central vision (2 m away at eye level). All subjects performed six trials in each experimental condition. Each trial lasted 30 s and was divided into two intervals of 15 s. During the first interval, sensory information (vision and/or proprioception) was occluded (vision) or masked (ankle proprioception). In the second interval, sensory information (vision and/or ankle proprioception) was given back; participants opened their eyes and/or the vibrations stopped (Fig. 1). This procedure allowed examining the *immediate *effect of the change in the availability of sensory inputs on balance control. The different sensory transition conditions were: i) reintegration of vision under normal proprioception (RV-P): i.e. no-vision/proprioception to vision/proprioception, ii) reintegration of proprioception under vision (RP-V): i.e. perturbed proprioception/vision (PP-V) to proprioception/vision (RP-V) and iii) reintegration of proprioception without vision (RP-NV): i.e. perturbed proprioception/no-vision (PP-NV) to proprioception/no-vision (RP-NV). The participant's balance control capability was also evaluated in trials without sensory manipulation where subjects kept their eyes opened in absence of ankle tendon vibration for 30 s (control condition). The experimental conditions were randomized within the experimental session and across participants.

### Data analysis

The CP data were filtered using a low-pass filter (Butterworth, 4^th ^order, 8 Hz cut-off frequency) with a dual pass to remove phase shift. The medio-lateral and antero-posterior velocities of the CP were calculated using finite difference technique. To characterize balance control, we calculated the root mean square (RMS) of the CP velocity along both axes. This parameter measures the variability of the CP sway path velocity. It is the square root of the sum of squares of the CP velocity divided by the number of data samples.

To study the mechanisms causing more variable CP velocity during sensory transition, we analyzed the sway density curve following Baratto et al. [44]. The sway density curve is computed by counting the number of consecutive samples during which the CP remains inside a 3 mm radius. Then, the sample count is divided by the sampling rate yielding a time dimension for the ordinate axis. Thus, the sway density curve is a time versus time curve illustrating the evolution over time of the *stay time *of the CP. The sway density curve was digitally filtered with a fourth-order Butterworth filter (2.5 Hz low pass cut-off frequency with dual-pass to remove phase shift) to perform a better peak extraction. The peaks of the sway density curve correspond to time instants in which the CP and presumably the associate motor commands are relatively stable. Mean peak represents the time spent by the CP inside the 3 mm radius circle centered at the time of peak on the sway density curve. Hence, the amplitude of the peaks estimates the variability of the balance control commands. On the other hand, the valleys of the sway density curve correspond to time instants in which the CP rapidly switches from one stable position to another. It is assumed that mean distance between consecutive peaks illustrates the amplitude of the balance control commands. Recently, Jacono et al. [61] have demonstrated that the centre of pressure displacement tends to be stable when the ankle torque is approximately constant, and this corresponds to peaks in the sway density curve. On the contrary, the centre of pressure tends to shift quickly when the ankle torque has strong peaks, and this corresponds to valleys in the sway density curve (see page 303 – Jacono et al., [61]). According to Baratto et al., (2002), mean peak (mean of the peaks of the sway density curve) and mean distance (mean distance between peaks of the sway density curve) are related to the capacity of the balance control system to integrate the sensory information and anticipate physiological internal delays to keep the vertical alignment of the whole body. We hypothesized that greater CP RMS velocity observed for scoliosis patients could be related to greater ankle torque commands (i.e. greater mean distance) and/or greater variability of the ankle torque commands (i.e. smaller mean peaks).

### Statistical design

To verify our hypothesis, both groups were compared before and after sensory transitions. The CP performance (RMS velocity and parameters related to the sway density curve) of the last 5 s of the sensory deprivation interval was compared with that of the first and last 5 s of the sensory reintegration interval. The first 5 s of the sensory deprivation interval had no particular interest here because we wanted to compare the specific effects of sensory reintegration on balance control. This 5 s was not considered in the analyses. By comparing balance control capability during the first and last 5 s of the sensory reintegration interval, we could evaluate the rapidity of the participants to re-weight the different sensory inputs. Analyses of variance (ANOVA) with repeated measures were used for statistical comparisons. For the control condition, although it did not involve any sensory transition, the same three 5 s epochs were used for comparisons. Post-hoc analyses were performed using Tukey method (*p *< 0.05).

## Results

### Balance stability for the control condition

**Figure 2:**
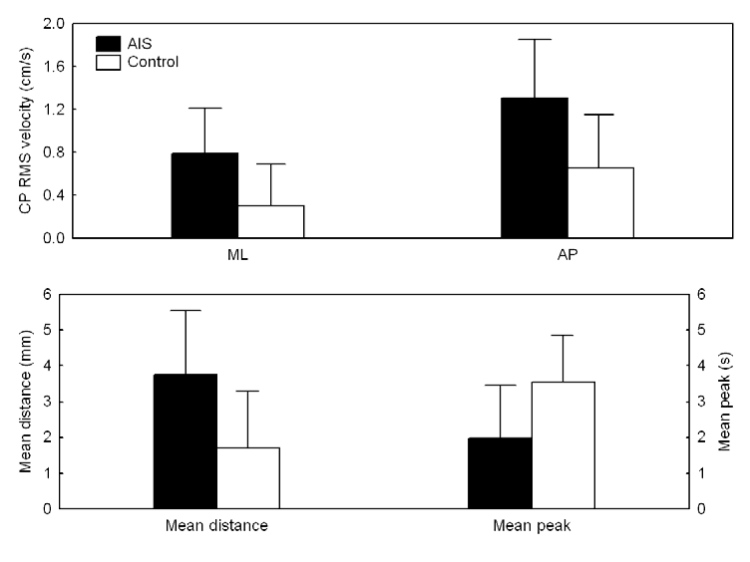
**Balance control performance in absence of sensory inputs manipulation**. Upper panel – Group means for the CP RMS velocity along the medio-lateral (ML) and antero-posterior (AP) axes for the control condition. Lower panel – Groups means of the distance between consecutive zones of stability (mean distance – left vertical axis) and the time spent within the zones of stability (mean peak – right vertical axis) for the control condition. On both panels, the error bars indicate 95% confidence intervals.

To quantify the baseline balance stability of AIS patients and control participants, the CP RMS velocity along the antero-posterior and medio-lateral axes were analyzed for the control condition (Fig. 2 – upper panel). The analysis showed main effects of Group [F_1,16 _= 12.82, *p *< 0.01] and Axis [F_1,16 _= 25.16, *p *< 0.001]. For both axes, AIS patients showed greater CP RMS velocity and this result was larger for the AP than for the ML axis. The main effect of Epoch was not significant [F_2,32 _= 2.95, *p *> 0.05]. No significant interactions between any of the independent variables (i.e., Group, Axis and Epoch) were found (*ps *> 0.05).

The analysis of the sway density curve (Fig. 2 – lower panel) revealed that in absence of sensory manipulation, AIS patients balance control commands were greater and more variable than those of control participants (main effects of Group: [F_1,16 _= 9.97, *p *< 0.01] and [F_1,16 _= 8.43, *p *< 0.05] for mean distance and mean peak, respectively).

### Balance stability for the reintegration of vision

**Figure 3:**
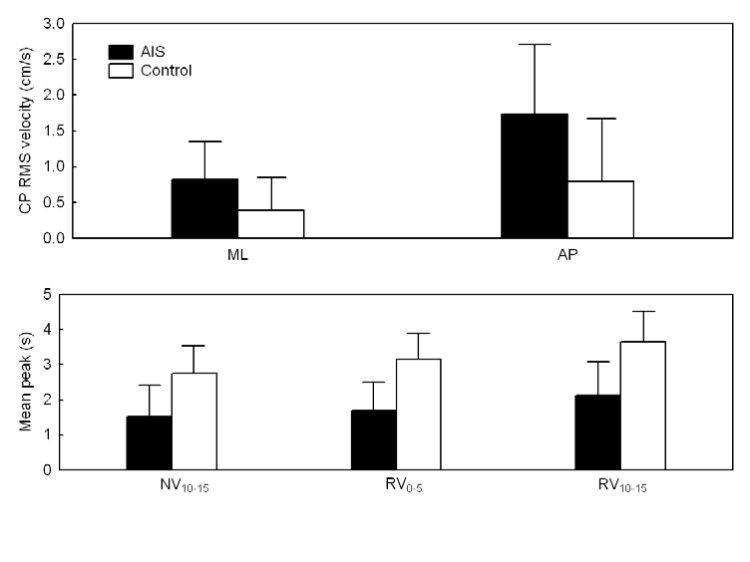
**Balance control performance during reintegration of vision**. Upper panel – Group means for the CP RMS velocity along the medio-lateral (ML) and antero-posterior (AP) axes for the proprioception/no-vision to proprioception/reintegration vision condition. Lower panel illustrates the interaction Group by Epoch for the time spent within the zones of stability (mean peak) for the last 5 s epoch of the proprioception/no vision interval (NV_10–15_) and first 5 s epoch (RV_0–5_) and last 5 s epoch (RV_10–15_) of the proprioception/vision reintegration intervals. On both panels, the error bars indicate 95% confidence intervals.

The analysis for the CP RMS along both axes (Fig. 3 – upper panel) during reintegration of vision showed main effects of Group [F_1,16 _= 7.32, *p *< 0.05], Axis [F_1,16 _= 25.56, *p *< 0.001] and Epoch [F_2,32 _= 4.16, *p *< 0.05]. AIS patients had greater CP RMS velocity than controls along both axes. The Axis by Epoch interaction was significant [F_2,32 _= 3.59, *p *< 0.05]. All other interactions were not significant (*ps *> 0.05).

The analysis of the sway density curve, time spent within the zones of stability (Fig. 3 – lower panel), revealed that the balance control commands of AIS patients were much more variable than that of controls (main effect of Group: [F_1,16 _= 6.66, *p *< 0.05]) regardless of the epoch (no Group × Epoch interaction: [F_2,32 _= 0.44, *p *> 0.05]). The main effect of Epoch was significant [F_2,32 _= 17.32, *p *< 0.001]. Post-hoc analysis demonstrated that both groups spent more time within the zones of stability when vision had returned to normal for at least 10 s; mean peak for the last epoch of the sensory reintegration interval (RV_10–15_) was larger than for the two other epochs (NV_10–15 _and RV_0–5_, *ps *< 0.01).

The analysis of the distance between two consecutive zones of stability (not illustrated) showed that, for both groups, the amplitude of the balance control commands were similar (no main effect of Group: [F_1,16 _= 3.62, *p *> 0.05] and no Group by Epoch interaction: [F_2,32 _= 2.65, *p *> 0.05]). Finally, the distance between zones of stability did not change when vision returned to normal (no main effect of Epoch: [F_2,32 _= 0.24, *p *> 0.05]).

### Balance stability for the reintegration of proprioception when vision was available

**Figure 4:**
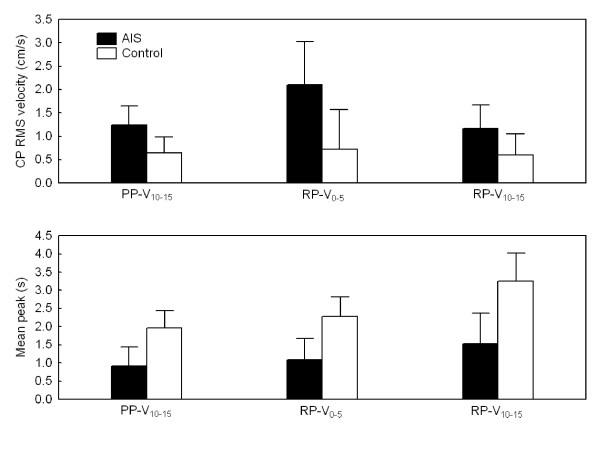
**Balance control performance during reintegration of ankle proprioception when vision is available**. Upper panel illustrates the interaction Group by Epoch for the CP RMS velocity for the last 5 s epoch of sensory deprivation internal (PP-V_10–15 _perturbed proprioception/vision) and for the first and last 5 s epoch of the sensory reintegration interval (RP-V_0–5 _and RP-V_10–15 _reintegration proprioception/vision). Lower panel shows the interaction Group by Epoch for the time spent within the zone of stability for the same 5 s epochs as the upper panels. On all panels, the error bars correspond to 95% confidence intervals.

The analysis of the RMS of the CP velocity (Fig. 4 – upper panel) revealed main effects of Group [F_1,16 _= 11.83, *p *< 0.01], Axis [F_1,16 _= 31.34, *p *< 0.001] and Epoch [F_2,32 _= 9.10, *p *< 0.001]. The interactions Group by Epoch [F_2,32 _= 5.70, *p *< 0.01] and Axis by Epoch [F_2,32 _= 4.36, *p *< 0.05] were significant. All other interactions were not significant (*ps *> 0.05). The decomposition of the Group by Epoch interaction indicated that during reintegration of ankle proprioception (PP-V_10–15 _versus RP-V_0–5_) the CP RMS velocity of scoliosis patients increased (p < 0.01) while it did not for controls (p > 0.05). Across time (RP-V_0–5 _versus RP-V_10–15_), however, scoliosis patients were able to decrease their CP RMS velocity (p < 0.01).

The analysis of the time spent within the zones of stability (Fig. 4 – lower panel) showed main effects of Group [F_1,16 _= 11.43, *p *< 0.005] and Epoch [F_1,16 _= 26.02, *p *< 0.001] and a significant interaction of Group by Epoch [F_2,32 _= 3.49, *p *< 0.05]. The decomposition of the interaction revealed that during reintegration of ankle proprioception (RP-V_0–5 _versus RP-V_10–15_) the time spent within the zones of stability increased for both groups (*p *> 0.05 and *p *< 0.001 for scoliosis patients and controls, respectively) but at the end of the sensory reintegration interval (RP-V_10–15_), AIS patients had more variable balance control commands (i.e. smaller mean peak values) than control participants (*p *< 0.01).

The ANOVA for the distance between consecutive zones of stability (i.e. mean distance; not illustrated) showed main effects of Group [F_1,16 _= 10.00, *p *< 0.01] and Epoch [F_2,32 _= 11.72, *p *< 0.001]. No significant interaction between Group and Epoch was found [F_2,32 _= 2.59, *p *> 0.05]. The decomposition of the main effect of Epoch illustrated that both groups had lower mean distance at the end of the sensory reintegration interval (last 5 s epoch: RP-V_10–15_) than immediately following sensory reintegration (first 5 s epoch: RP-V_0–5_).

### Balance stability for the reintegration of proprioception without vision

**Figure 5:**
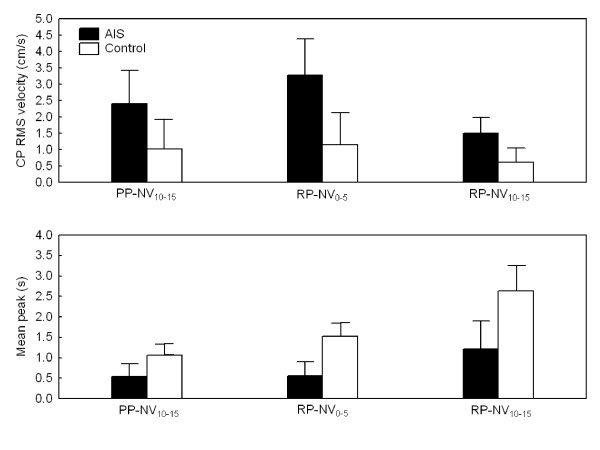
**Balance control performance during reintegration of ankle proprioception in absence of vision**. Upper panel presents the interaction Group by Epoch for the CP RMS velocity for the last 5 s epoch of sensory deprivation interval (PP-NV_10–15 _perturbed proprioception/no-vision) and for the first and last 5 s epoch of the sensory reintegration interval (RP-NV_0–5 _and RP-NV_10–15 _reintegration proprioception/no-vision condition). Lower panel illustrates the interaction Group by Epoch for the time spent within the zone of stability for the same 5 s epochs as the upper panels. On all panels, the error bars indicate 95% confidence interval.

The analysis of the RMS of the CP (Fig. 5 – upper panel) indicated main effects of Group [F_1,16 _= 19.99, *p *< 0.001], Axis [F_1,16 _= 5.00, *p *< 0.05] and Epoch [F_2,32 _= 16.15, *p *< 0.001]. The interaction Group by Epoch revealed significant [F_2,32 _= 4.69, *p *< 0.05]. All other interactions were not significant (*ps *> 0.05).

The analysis of the sway density curve (Fig. 5 – lower panel) revealed main effects of Group [F_1,16 _= 13.11, *p *< 0.01], Epoch [F_2,32 _= 42.91, *p *< 0.001] and a significant interaction of Group by Epoch [F_2,32 _= 6.38, *p *< 0.01]. The decomposition of the interaction indicated that the balance control commands of AIS patients was still more variable during the first 5-s of the sensory reintegration interval (RP-NV_0–5_) as their mean peak did not increase from PP-NV_10–15 _to RP-NV_0–5 _(*p *> 0.05). On the contrary, control participants reduced the variability of their balance control commands as their mean peak increased from PP-NV_10–15 _to RP-NV_0–5 _(*p *< 0.01).

The ANOVA for the distance between zones of stability (not illustrated) showed main effects of Group [F_1,16 _= 15.68, *p *< 0.01], Epoch [F_2,32 _= 21.27, *p *< 0.001] and a significant interaction of Group by Epoch [F_2,32 _= 6.24, *p *< 0.01]. The decomposition of the interaction suggested that when ankle proprioception returned to normal in absence of vision (PP-NV_10–15 _to RP-NV_0–5_), both groups did not reduce the distance between consecutive zones of stability (*ps *> 0.05). During the sensory reintegration interval, AIS patients reduced the mean distance to a greater extent than control participants (*p *< 0.01). At the end of the sensory reintegration interval, however, mean distance of AIS patients was still greater than that of control participants (*p *< 0.05) suggesting that the amplitude of the balance control commands of scoliosis patients were much greater.

## Discussion

This is an extension of previous work which assessed whether adding sensory inputs altered AIS balance control [31]. In this study, we determined the effect of sensory deprivation on balance control of idiopathic scoliosis patients. It has been shown that tendon vibration reduced the amplitude of short-latency responses but not the medium-latency responses after toe-up perturbation [45]. Following tendon vibration, however, the medium-latency responses decreased of about 42% whereas prompt recovery was observed in the short-latency responses. In contrast to short-latency responses, only the medium-latency responses have a stabilizing effect and it is influenced by 'postural set' [46]. These latter observations suggest that the mechanisms controlling balance during sensory deprivation and reintegration differ.

The present study provides evidence that adolescent idiopathic scoliosis patients, when sensory information is restored, have difficulty in dynamically adjusting the weight of the various sensory inputs to tailor the balance control commands to the mechanical context. For any given configuration of sensory information, the AIS group showed greater spontaneous CP velocity variability compared to healthy control participants. Similar results have been reported in the literature [30,40,47,48]. It has been suggested that balance control dysfunction in AIS patients could be the consequence of biomechanical factors such as the three-dimensional deviation of the spine; changes in the orientation of various segments [3,49]. The fact that scoliosis patients had greater CP RMS velocity than controls in absence of sensory manipulation acknowledges that spine deformity (*biomechanical hypothesis*) may exacerbate patients balance control problem. Remarkably, however, returning sensory inputs to normal perturbed patients balance. This suggests that the deficit is not only related to the three-dimensional deviation of the spine but also to a difficulty in dynamically adjusting the weight of the various sensory inputs (*sensory integration hypothesis*). This supports the result of O'Beirne et al [50]. In their experiment, seven patients with progressive curves underwent surgical correction and stabilization. Patients were tested preoperatively and six months postoperatively. The authors observed no improvement in balance control even though a curve reduction was reported.

The novelty of the present study is that AIS patients showed greater RMS CP velocity following reintegration of proprioception inputs from muscles acting at the ankle joint. This observation was present whether or not vision was available. In contrast, the control participants were able to maintain or rapidly reduce their CP velocity variability when proprioceptive information from the lower leg muscles returned to normal. Moreover, in absence of vision, the AIS group exhibited an increased RMS of their CP velocity following reintegration of ankle proprioception. The increase of CP RMS velocity observed in the context of sensory transition (i.e., sensory information return to normal; no tendon vibration and/or eyes open) indicates that, for the AIS patients, the central sensory-reweighting mechanisms are less effective. The greater RMS CP velocity observed during sensory reintegration suggests that AIS patients balance control commands were inappropriate. The analysis of their sway density plot showed that AIS patients had greater mean peak (balance control commands variability) and mean distance (balance control commands amplitude). This could result from an improper transformation of the sensory-orientation cues into corrective balance control commands. During tendon vibration, peripheral drive to the motoneurones could increase the efficacy of group II drive [45]. In this situation, it has been proposed that the CNS may reduce the central drive directed to the spinal cord, motoneurones or group II interneurones. Following tendon vibration, however, the central drive would need to return to its 'default' state and this process would take time [51]. Our results suggest that the mechanisms in charge of re-adapting the central drive following tendon vibration may respond more tardily in scoliosis patients than controls.

The reintegration of vision only, however, did not change the ability of AIS patients to control balance. It is likely that the contribution of proprioception was sufficient to select the appropriate corrective balance control commands (as suggested by the absence of an interaction Group by Epoch in the reintegration of vision condition). It is possible that, in this condition, both groups relied mainly on proprioceptive inputs to regulate their body sway oscillations hence, minimizing sensory-reweighting. The fact that the proprioceptive threshold is significantly lower than the visual and/or vestibular threshold during upright standing supports this hypothesis[52].

Idiopathic scoliosis may be related to peripheral sensory system impairments, spinal cord and/or central sensory integrative mechanism problems. A hypersensitivity to tendon vibration has been reported in AIS patients [37]. Byl et al. [30], however, have measured vibratory threshold at the cervical spine, wrist, and foot in AIS and age-matched control participants. The vibratory thresholds were similar in adolescent idiopathic scoliosis and their age-matched controls. Measurement of nerve conduction velocity in the peroneal and median nerves did not support the theory that polyneuropathy is involved in idiopathic scoliosis [53]. Concerning long-reflex activity, Maguire et al. [54] have observed ipsilateral and contralateral long-latency polysynaptic activity in all 37 adolescent idiopathic scoliosis patients they tested. Interestingly, this activity was absent in adolescent non-idiopathic scoliosis patients with spinal deformities of equal magnitude than that of AIS patients suggesting that the spinal curve per se is not responsible.

Other studies have suggested that idiopathic scoliosis could be related to neurological deficits associated with an organic affectation at the brainstem level. For instance, Yamamoto et al. [55] have observed a positive correlation between brainstem dysfunction, determined by visual target pursuit tests, and curve progression. They concluded that brainstem might be the structure which plays a major role in idiopathic scoliosis progression. Besides, results from animal studies have demonstrated reticulospinal connections with axial muscles as stimulation of the medullary reticular formation evoked deep lumbar back muscles activity and lesion of this site decreases lordosis performance [56-59]. Others have observed that lateral gaze palsy is associated with a high prevalence of idiopathic scoliosis suggesting that the site of neurological abnormality might be the paramedian pontine reticular formation [60].

## Conclusion

Altogether, the present results demonstrate that idiopathic scoliosis adolescents have difficulty in reweighting sensory inputs following a brief period of sensory deprivation.

## Competing interests

The author(s) declare that they have no competing interests.

## Authors' contributions

MS, NT and PA conceived the study. PM recruited subjects and examined all patients. MS, JB and NT evaluated the data, performed data analyses and wrote the manuscript. All authors read and approved the final manuscript
